# DNA methylation of microRNA‐coding genes in non‐small‐cell lung cancer patients

**DOI:** 10.1002/path.5079

**Published:** 2018-06-20

**Authors:** Gerwin Heller, Corinna Altenberger, Irene Steiner, Thais Topakian, Barbara Ziegler, Erwin Tomasich, György Lang, Adelheid End‐Pfützenreuter, Sonja Zehetmayer, Balazs Döme, Britt‐Madeleine Arns, Walter Klepetko, Christoph C Zielinski, Sabine Zöchbauer‐Müller

**Affiliations:** ^1^ Department of Medicine I, Clinical Division of Oncology Medical University of Vienna Vienna Austria; ^2^ Comprehensive Cancer Centre Medical University of Vienna Vienna Austria; ^3^ Centre for Medical Statistics, Informatics and Intelligent Systems, Section for Medical Statistics Medical University of Vienna Vienna Austria; ^4^ Department of Thoracic Surgery Medical University of Vienna Vienna Austria; ^5^ Department of Thoracic Surgery National Institute of Oncology‐Semmelweis University Budapest Hungary; ^6^ Department of Tumour Biology National Koranyi Institute of Pulmonology Budapest Hungary; ^7^ Landesklinikum Thermenregion Hochegg Grimmenstein Austria

**Keywords:** CpG island methylation, miRNA, MeDIP‐chip, MS‐HRM analysis, non‐small‐cell lung cancer

## Abstract

Deregulated DNA methylation leading to transcriptional inactivation of certain genes occurs frequently in non‐small‐cell lung cancers (NSCLCs). As well as protein‐coding genes, microRNA (miRNA)‐coding genes may be targets for methylation in NSCLCs; however, the number of known methylated miRNA genes is still small. Thus, we investigated methylation of miRNA genes in primary tumour (TU) samples and corresponding non‐malignant lung tissue (NL) samples of 50 NSCLC patients by using methylated DNA immunoprecipitation followed by custom‐designed tiling microarray analyses (MeDIP‐chip), and 252 differentially methylated probes between TU samples and NL samples were identified. These probes were annotated, which resulted in the identification of 34 miRNA genes with increased methylation in TU samples. Some of these miRNA genes were already known to be methylated in NSCLCs (e.g. those encoding miR‐9‐3 and miR‐124), but methylation of the vast majority of them was previously unknown. We selected six miRNA genes (those encoding miR‐10b, miR‐1179, miR‐137, miR‐572, miR‐3150b, and miR‐129‐2) for gene‐specific methylation analyses in TU samples and corresponding NL samples of 104 NSCLC patients, and observed a statistically significant increase in methylation of these genes in TU samples (p < 0.0001). In silico target prediction of the six miRNAs identified several oncogenic/cell proliferation‐promoting factors (e.g. CCNE1 as an miR‐1179 target). To investigate whether miR‐1179 indeed targets CCNE1, we transfected miR‐1179 gene mimics into CCNE1‐expressing NSCLC cells, and observed downregulated CCNE1 mRNA expression in these cells as compared with control cells. Similar effects on cyclin E1 expression were seen in western blot analyses. In addition, we found a statistically significant reduction in the growth of NSCLC cells transfected with miR‐1179 mimics as compared with control cells. In conclusion, we identified many methylated miRNA genes in NSCLC patients, and found that the miR‐1179 gene is a potential tumour cell growth suppressor in NSCLCs. Overall, our findings emphasize the impact of miRNA gene methylation on the pathogenesis of NSCLCs. © 2018 The Authors. *The Journal of Pathology* published by John Wiley & Sons Ltd on behalf of Pathological Society of Great Britain and Ireland.

## Introduction

MicroRNAs (miRNAs) are short (∼22 nucleotides in length), non‐coding RNAs that act as post‐transcriptional regulators of gene expression 1. So far, in humans, >1800 miRNAs have been identified, and many of them are involved in the regulation of biological processes including cellular differentiation, proliferation, and apoptosis 2. It has been shown that deregulated expression of certain miRNAs may lead to alterations in these processes and to the development of a malignant phenotype 3. Downregulated expression of numerous miRNAs in primary tumours of non‐small‐cell lung cancer (NSCLC) patients was found when miRNA expression patterns in primary tumour (TU) samples and matching non‐malignant lung tissue (NL) samples of these patients were compared by the use of microarray analyses 4, 5, 6. DNA methylation (referred to as methylation) was identified as a mechanism that may cause downregulation of miRNA gene expression in cancer cells 7, 8.

Methylation is part of the epigenetic gene regulation machinery, and involves the covalent addition of a methyl group to the 5′ carbon of cytosine within cytosine–guanosine (CG) dinucleotides 9. Although CG dinucleotides are relatively rare in the mammalian genome, certain genomic regions, called CpG islands (CGIs), contain CG dinucleotides at a high density 9. CGIs are found in ∼60% of the human gene promoter regions, including both protein‐coding and miRNA‐coding genes 10, 11. Methylation was found to be a reversible change, and DNA methyltransferase inhibitors, e.g. 5‐aza‐2′‐deoxycytidine (Aza‐dC), may act synergistically with histone deacetylase inhibitors, e.g. trichostatin A (TSA), on gene re‐expression 12. In NSCLCs, numerous tumour suppressor genes (TSGs) have been found that are frequently methylated, and thus transcriptionally silenced 13, 14, 15, 16. Moreover, several miRNA genes that are transcriptionally regulated by methylation (e.g. those encoding miR‐34 family members, miR‐124a, miR‐126, miR‐9‐3, and miR‐193a) have been identified in NSCLCs 8, 17, 18, 19, 20.

Because knowledge about methylation‐mediated miRNA silencing in NSCLCs is still limited, we performed a microarray‐based screen for methylated miRNA genes in TU samples and corresponding NL samples of 50 NSCLC patients by combining methylated DNA immunoprecipitation and custom‐designed tiling microarray analyses (MeDIP‐chip). MeDIP‐chip results were confirmed by the use of gene‐specific approaches in a large cohort of NSCLC patients. The methylation and expression of selected miRNA genes before and after treatment of NSCLC cells with epigenetically active drugs were analysed. In addition, the so far functionally uncharacterized miR‐1179 was selected to be investigated for potential tumour cell growth‐suppressing properties. Finally, miRNA methylation data were compared with clinicopathological characteristics of the NSCLC patients.

Overall, we identified many miRNA genes with increased methylation in TU samples as compared with NL samples of NSCLC patients, and demonstrated that methylation of certain miRNA genes is associated with transcriptional gene regulation; we suggest that miR‐1179 might be a novel tumour cell growth suppressor in NSCLCs.

## Materials and methods

### Tissue samples and tumour cell lines

This study was approved by the local ethics committee. For methylation analyses, we used patient‐matched TU samples and NL samples of Caucasian NSCLC patients who underwent surgical resection of their tumour with curative intent during the years 2000–2004 21. Tissue samples were snap‐frozen and stored in liquid nitrogen until use. Quality assessment of samples for appropriateness, diagnosis of NSCLC and histological subtyping was performed by certified pathologists on formalin‐fixed paraffin‐embedded sections according to standard criteria; however, no re‐evaluation was performed before the beginning of this study. The tumour cell content of the tumour specimens was at least 50%. MeDIP‐chip analyses and methylation‐sensitive high‐resolution melting (MS‐HRM) analyses were performed on TU samples and NL samples from 50 and 104 patients, respectively. Clinicopathological data of these patients are shown in Table 1 and supplementary material, Table S1. Disease‐free survival (DFS) and overall survival (OS) data were available for 88 patients.

**Table 1:** Clinicopathological characteristics of 104 NSCLC patients

Variables	*N*
Age (years)	
<60	48
≥60	56
Gender	
Male	58
Female	46
Histology^a^	
Adenocarcinoma	69
Squamous cell carcinoma	35
Disease stage	
I	41
II	30
III	31
IV	2
T stage	
T1	20
T2	56
T3	24
T4	4
N stage	
N0	52
N1	26
N2	23
N3	1
NX	2
Disease recurrence	
No	77
Yes	27

Median age, 60 years; median follow‐up, 60 months.

a See 21.

The authenticated NSCLC cell lines A549, NCI‐H1650, NCI‐H1975, NCI‐H1993 and NCI‐H2073 were purchased from the American Type Culture Collection (Manassas, VA, USA) and were stored in liquid nitrogen until use. Genomic DNA was isolated from tissue samples and tumour cell lines by digestion with proteinase K, followed by standard phenol/chloroform extraction and ethanol precipitation, and was stored at –80 °C until use. Treatment of cells with epigenetically active drugs was performed as reported previously 22.

### MeDIP‐chip analyses

A detailed description of MeDIP‐chip analyses is provided in supplementary material, Supplementary materials and methods.

### MS‐HRM analysis

Genomic DNA was modified by treatment with sodium bisulphite 8. Primer sequences were designed with Methyl Primer Express v.1.0 software (supplementary material, Table S2). An EpiTect HRM polymerase chain reaction (PCR) kit in a RotorGene Q cycler (Qiagen, Hilden, Germany) was used. Methylation standards were constructed by diluting 100% methylated and unmethylated control DNA (Qiagen) at 100%, 75%, 50%, 25%, 10% and 0% ratios 16, 23. Normalized fluorescence values were plotted against the percentage of methylation for each standard to generate a standard curve for the calculation of methylation levels of genes in patient samples. Water blanks were used as negative controls.

### Transfection experiments

Cells were transfected with miRNA mimics, random sequence miRNA mimic controls or miRNA inhibitors (4464066_MC13164, 4464058, 4464084_MH13164; Ambion, Carlsbad, CA, USA) by the use of Lipofectamine RNAiMax Reagent (Invitrogen, Carlsbad, CA, USA). In brief, 4 × 10^5^ cells/well were seeded in a six‐well plate, followed by transfection of 200 nm miRNA mimics or controls. Total RNA was isolated after 48 h. Stable transfection of NSCLC cells was performed with an miRNA‐1179‐expressing plasmid (SC400028; Origene, Rockville, MD, USA), a Lipofectamine 3000 Transfection kit (Invitrogen), and 500 μg/ml G418.

### Reverse transcription PCR

RNA was isolated with RNeasy and miRNeasy Kits (Qiagen). cDNA synthesis was performed with total RNA from NSCLC cells by use of the miScript II RT Kit (Qiagen). Levels of mRNA from selected genes were quantified with TaqMan reverse transcription PCR (RT‐PCR) assays (Hs01026536_m1, Hs00866536_s1, Hs00388292_m1, Hs00270274_m1, Hs0275899_g1; ABI, Carlsbad, CA, USA) and the ABI Step One Plus Detection System (ABI, Carlsbad, CA, USA), according to the manufacturer's protocol. Fold changes in expression were calculated with the ΔΔCt method. To quantify the expression of selected miRNAs, RT‐PCR with the QuantiTect SYBR Green PCR Kit (Qiagen) was performed. The following miScript Primer Assays (Qiagen) were used: MS00014084 (miR‐1179 gene) and MS00003682 (reference, miR‐191 gene).

### Western blotting

Total proteins were isolated by use of a RIPA lysis buffer supplemented with cOmplete™ protease‐inhibitor cocktail (Roche, Mannheim, Germany). Aliquots (20 μg) of protein were separated in a 10% polyacrylamide gel, blotted onto a poly(vinylidene difluoride) membrane, and blocked for 1 h in 5% non‐fat dry milk. The membrane was incubated overnight with primary antibodies (anti‐mouse α‐tubulin, 1:100, #T9026; anti‐rabbit cyclin E1, 1:500, #SAB4503514; Sigma‐Aldrich, St Louis, MO, USA) diluted in 5% milk. This was followed by washing with phosphate‐buffered saline supplemented with Tween‐20 (PBS‐T). The membrane was then incubated with horseradish peroxidase‐conjugated secondary antibodies (sc‐2004 and sc‐2005, 1:1000; Santa Cruz Biotechnology, Santa Cruz, CA, USA) for 90 min, washed with PBS‐T, and developed with the Clarity Western ECL substrate (Bio‐Rad, Richmond, CA, USA). Membranes were stripped between the antibodies with Restore Western Blot Stripping Buffer (Thermo Fisher Scientific, Waltham, MA, USA). Images were captured with the ChemiDoc XRS device (Bio‐Rad).

### Chromatin immunoprecipitation (ChIP) assay

ChIP assays were performed with the Magna Chip A kit (Millipore, Bedford, MA, USA), a Bioruptor (Diagenode, Liege, Belgium), and the following antibodies: anti‐acetyl‐histone H3 (#06‐599; 1:100; Millipore), anti‐acetyl‐histone H4 (#06‐866; 1:100; Millipore), and normal rabbit IgG (2729S; 1:100; Cell Signaling, Frankfurt, Germany). Real‐time quantitative PCR (qPCR) was performed with GoTaq qPCR Master Mix (Promega, Madison, WI, USA) and 2 μl of sample per reaction. ChIP‐qPCR data were analysed with the percentage input method 24.

### Cell viability and proliferation assays

Cell viability was determined by use of a CellTiter‐Blue Cell Viability Assay (Promega). Cells were plated in triplicate in 96‐well plates, and incubated with CellTiter‐Blue Reagent (Promega) prior to fluorescence measurement. Cell proliferation was measured in real time with the xCELLigence Real‐Time Cellular Analysis system (Roche). At 24 h after transfection with miR‐1179 mimics/inhibitors, cells were seeded in triplicate in 16‐well E‐plates, and cell proliferation was monitored for 1 week.

### 3′‐Untranslated region (UTR) reporter assay

Cells were cotransfected with a luciferase reporter construct containing the 3′‐UTR of *CCNE1* (SC207262; Origene, Rockville, MD, USA) and with miRNA‐1179 mimics (4464066_MC13164; Ambion) or random sequence miRNA mimic controls. Interactions between miRNAs and targets were measured with the Britelite plus luminescence reporter assay system (PerkinElmer, Boston, MA, USA). Firefly luciferase was used as the primary reporter to monitor mRNA regulation. To confirm the specificity of miRNA–mRNA interaction, cells were cotransfected with the same luciferase reporter construct lacking the miRNA‐1179 seed sequence in the 3′‐UTR of *CCNE1*.

### Publicly available datasets

RNA‐sequencing (RNA‐seq) data and clinicopathological data of the LUAD (lung adenocarcinoma) and LUSC (lung squamous cell carcinoma) datasets were obtained from database of the The Cancer Genome Atlas (TCGA) (http://cancergenome.nih.gov), from cBioPortal for Cancer Genomics (http://www.cbioportal.org), and from Cancer Browser (https://genome-cancer.ucsc.edu/) 25.

### Statistical analyses

A detailed description of all statistical methods used in this study is provided in supplementary material, Supplementary materials and methods.

## Results

### Comparison of miRNA methylation microarray data from TU samples and NL samples

**Figure 1:**
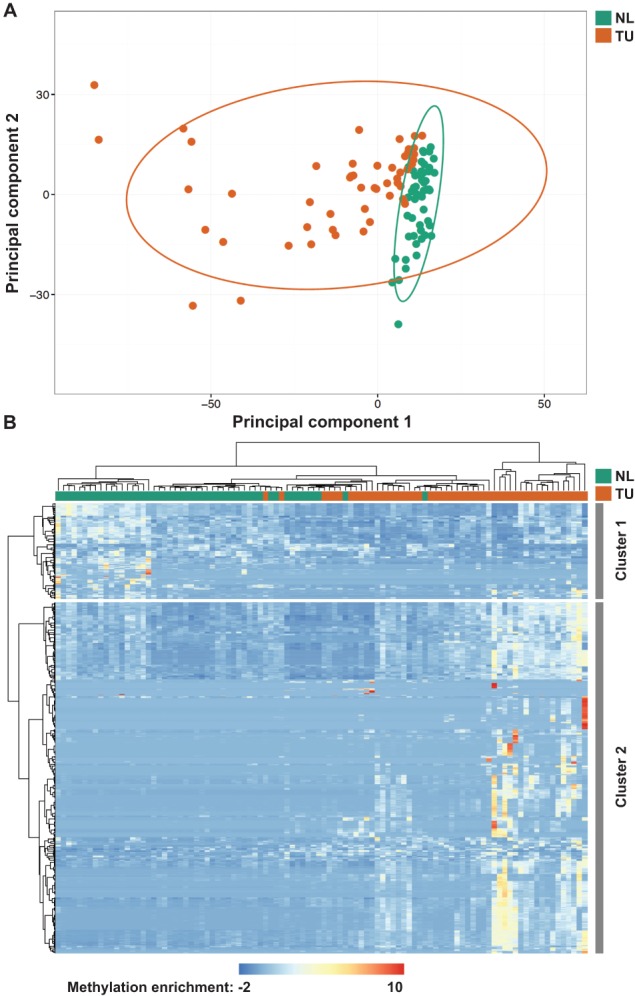
Identification of differentially methylated miRNA genes in NL samples and TU samples of 50 NSCLC patients by MeDIP‐chip analyses. (A) Results from principal component analyses of NL samples (green) and TU samples (orange) based on statistically significantly enriched microarray probes. (B) The heatmap illustrates differential methylation of miRNA genes in TU samples and NL samples. Overall, 1477 probes representing differential methylation of 252 unique genomic positions are shown. Colours range from blue (low methylation) to red (high methylation). Cluster 1 represents miRNA genes with increased methylation in NL samples as compared with TU samples. Cluster 2 represents miRNA genes with increased methylation in TU samples as compared with NL samples.

We performed custom‐designed MeDIP‐chip analyses and investigated the methylation of miRNA genes in TU samples and NL samples of 50 NSCLC patients (GEO accession no. GSE86173). For each of the samples, raw data representing signal intensities of Cy5‐labelled methylation‐enriched DNA and Cy3‐labelled input DNA were generated for 574 392 probes. Raw microarray data were normalized (supplementary material, Figure S1), and signal intensities of all probes from TU samples and NL samples were compared. After correcting for multiple testing, we found significant differences (family‐wise error rate of <0.1) at 252 genomic positions. Methylation of these positions clearly distinguished TU samples from NL samples (Figure 1A). Whereas 195 of the differentially methylated probes were found to be methylated to a statistically significantly higher extent in TU samples than in NL samples (referred to as increased methylation in TU samples), 57 of them were methylated to a statistically significantly higher extent in NL samples than in TU samples (referred to as increased methylation in NL samples) (Figure 1B). Differentially methylated probes were annotated (UCSC Genome Browser, effective May 2015), resulting in the identification of 34 unique miRNA genes with increased methylation in TU samples, and in 15 miRNA genes with increased methylation in NL samples (composite from all 50 NSCLC patients). Genomic sequences of miRNA genes with increased methylation in TU samples were obtained from the ENSEMBL database (release 90), and were used for a CGI search. Overall, 27 of the 34 (79%) miRNA genes were found to be associated with a CGI. Detailed information on these genes is shown in supplementary material, Tables S3 and S4. Only for some of them (the miR‐9‐3, miR‐124‐1/2 and miR‐129‐2 genes) regulation by methylation in NSCLCs has been described previously 8, 17, 26. For the majority of the miRNA genes, it was previously unknown whether they are methylated in NSCLC patients.

### MS‐HRM analyses of selected miRNA genes

**Figure 2:**
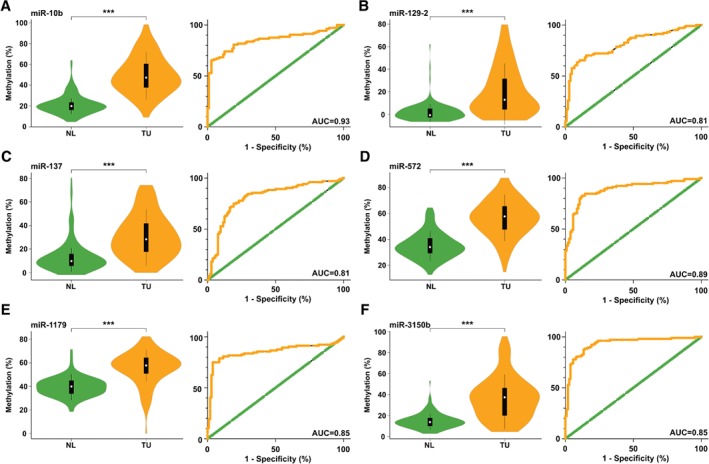
MS‐HRM analyses of the (A) miR‐10b, (B) miR‐129‐2, (C) miR‐137, (D) miR‐572, (E) miR‐1179 and (F) miR‐3150b genes in TU samples and NL samples from 104 NSCLC patients. Statistically significant differences in miRNA methylation between TU samples and NL samples were found, and are summarized by violin plots. In addition, ROC curves demonstrating the discrimination between TU samples and NL samples of 104 NSCLC patients on the basis of miRNA methylation are shown. ***p < 0.0001.

We developed gene‐specific MS‐HRM assays to investigate the methylation of selected miRNA genes (those encoding miR‐10b, miR‐129‐2, miR‐137, miR‐572, miR‐1179, and miR‐3150b) in TU samples and NL samples of 104 NSCLC patients in total (supplementary material, Figure S2). Consistent with our MeDIP‐chip data, we observed a statistically significant increase in methylation in TU samples of all miRNA genes investigated with MS‐HRM analyses (p < 0.0001; Figure 2A–F). In addition, receiver operating characteristic (ROC) curve analyses revealed that methylation of all miRNA genes analysed statistically significantly distinguished TU samples from NL samples [area under the curve (AUC) range, 0.81–0.93; Figure 2A–F].

Moreover, for each patient, TU/NL methylation ratios of the six miRNA genes were calculated, and patients with a TU/NL ratio of ≥1.5 were considered to be methylated for these miRNA genes (supplementary material, Table S5) 8. The most frequently methylated miRNA gene was that encoding miR‐10b (82%), followed by those encoding miR‐3150b (71%), miR‐137 (70%), miR‐572 (61%), miR‐129‐2 (61%), and miR‐1179 (40%). Methylation data for the six miRNA genes were compared with the clinicopathological characteristics of our NSCLC patients, including gender, age, histology, tumour stage, lymph node stage, stage of disease, disease recurrence, DFS, and OS. The percentage of miR‐129‐2 gene methylation was statistically significantly higher in T3 tumours than in T1 tumours (*p* = 0.032), and in TU samples from stage III patients than in TU samples from stage I/stage II patients (*p* = 0.038 and *p* = 0.048, respectively). In addition, miR‐1179 gene methylation was higher in female than in male patients (*p* = 0.04). No statistically significant associations regarding DFS and OS were found. A list of all comparisons is shown in supplementary material, Table S6.

### miRNA target prediction and gene ontology (GO) analyses of predicted miRNA targets

**Figure 3:**
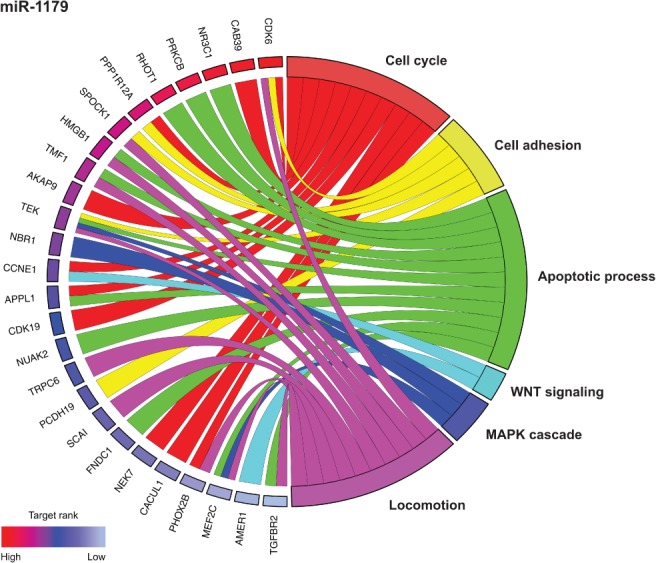
The top 30 predicted targets of miR‐1179 and their relationships with certain molecular pathways. Targets are ranked, on the basis of their prediction score, from red (highest score) to light blue (lowest score).

To obtain information about potential targets of miR‐10b, miR‐129‐2, miR‐137, miR‐572, miR‐1179, and miR‐3150b, we performed *in silico* miRNA target prediction followed by GO analyses for each of these miRNAs. By miRNA target prediction, we identified 53 target genes for miR‐10b, 85 for miR‐129‐2, 212 for miR‐137, two for miR‐572, 84 for miR‐1179, and 96 for miR‐3150b (supplementary material, Table S7). Only a few of these genes were predicted targets of more than one miRNA analysed (supplementary material, Figure S3). GO analyses of predicted miRNA targets identified several molecular pathways whose deregulation may contribute to a malignant phenotype. Examples of miRNA‐1179 targets that were predicted to be involved in apoptosis (e.g. *NR3C1* and *HMGB1*), the cell cycle (e.g. *CDK6* and *CCNE1*), cell adhesion (e.g. *SPOCK1* and *PCDH19*), WNT signalling (e.g. *TLE4* and *CCNE1*), mitogen‐activated protein kinase (*MAPK*) signalling (e.g. *NBR1* and *MEF2C*) and locomotion (e.g. *TMF1* and *TEK*) are shown in Figure 3. Results of GO analyses of the other miRNA targets are shown in supplementary material, Figures [Link], [Link].

### Effect of miR‐1179 mimic expression on target mRNAs

**Figure 4:**
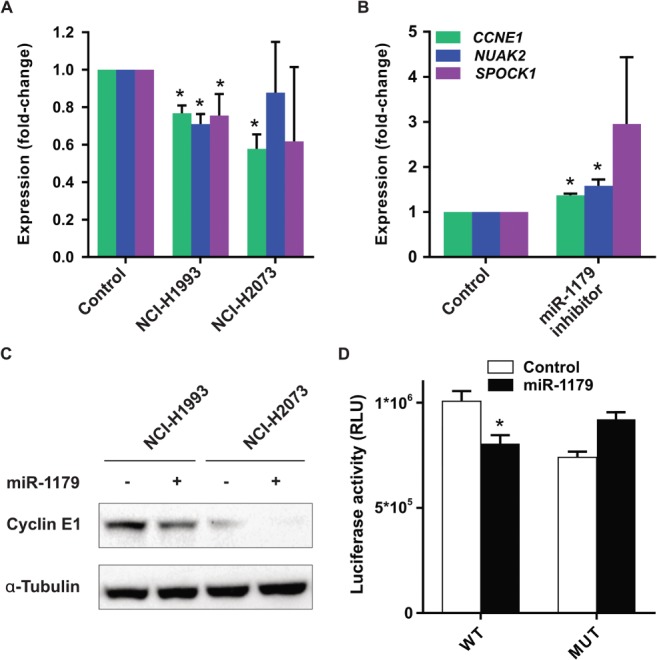
Predicted miR‐1179 targets in NSCLC cells and in NSCLC patients. (A) Expression of the predicted miR‐1179 targets CCNE1, NUAK2 and SPOCK1 was measured in miR‐1179 mimic‐transfected NCI‐H1993 and NCI‐H2073 cells. Fold changes relative to control cells are shown. *p < 0.05. (B) Stably transfected A549^pCMV‐miR‐1179^ cells were treated with an miR‐1179 inhibitor, and RT‐PCR showed upregulated expression of CCNE1, NUAK2 and SPOCK1. *p < 0.05. (C) Western blot analysis of cyclin E1 in NCI‐H1993 and NCI‐H2073 cells transfected with control RNA (–) or miR‐1179 (+). (D) Luciferase activity in cells that were cotransfected with a luciferase reporter construct containing either the wild‐type (WT) or the mutated (MUT) 3′‐UTR of CCNE1 and with miR‐1179 mimics. RLU, relative luminescence units.

Because the role of miR‐1179 in the pathogenesis of NSCLCs is currently unknown, we selected this miRNA for further experiments. We transfected NSCLC cells with miR‐1179 mimics to study its effect on expression of the predicted target genes CCNE1, NUAK2, and SPOCK1. Random sequence miRNA mimic‐transfected cells were used as controls. In NCI‐H1993 cells, all three targets were statistically significantly downregulated; however, in NCI‐H2073 cells, only CCNE1 downregulation reached statistical significance (Figure 4A). Upregulated CCNE1, NUAK2 and SPOCK1 expression was found in A549^pCMV‐miR‐1179^ cells after miR‐1179 inhibitor transfection (Figure 4B). Western blot analyses revealed that cyclin E1 expression was also downregulated in NCI‐H1993 and NCI‐H2073 cells at the protein level (Figure 4C). To confirm that miR‐1179 regulates CCNE1 expression, we performed a 3′‐UTR reporter assay, and detected reduced luciferase activity in cells transfected with miR‐1179 mimics as compared with control cells (Figure 4D). These results suggest that cyclin E1 is indeed regulated by miR‐1179 in these cell lines.

### Expression of the predicted miR‐1179 target CCNE1 in NSCLCs

Our GO analyses demonstrated that *CCNE1* is among the top‐ranked miR‐1179 targets. Because *CCNE1* is involved in cell cycle regulation, and its deregulation has been reported in cancer cells, we selected *CCNE1* for gene expression analyses and for functional *in vitro* assays 27, 28. We analysed *CCNE1* expression in TU samples (*N* = 1015) and NL samples (*N* = 109) of NSCLC patients from the two publicly available RNA‐seq datasets LUAD and LUSC of the TCGA database 29. Whereas the median *CCNE1* expression levels in TU samples were 7.88 and 6.8, respectively, the median *CCNE1* expression levels in NL samples were 4.22 and 3.78, respectively (supplementary material, Figure S9). The difference in *CCNE1* expression between TU samples and NL samples was statistically highly significant in both datasets (*p* < 0.0001). Comparison of *CCNE1* expression data with clinicopathological characteristics, including age, tumour stage, lymph node stage, and stage of disease, of patients from the LUAD and LUSC datasets did not reveal a statistically significant association. However, lung adenocarcinoma patients with low *CCNE1* expression had a statistically significant longer OS than those with high *CCNE1* expression (supplementary material, Figure S9C,D). Similar results were obtained in a second patient cohort for lung adenocarcinomas and lung squamous cell carcinomas (supplementary material, Figure S9E,F).

### Effect of epigenetically active drugs on miRNA‐1179 gene expression and effect of miR‐1179 gene expression on cell viability and cell proliferation of NSCLC cells

**Figure 5:**
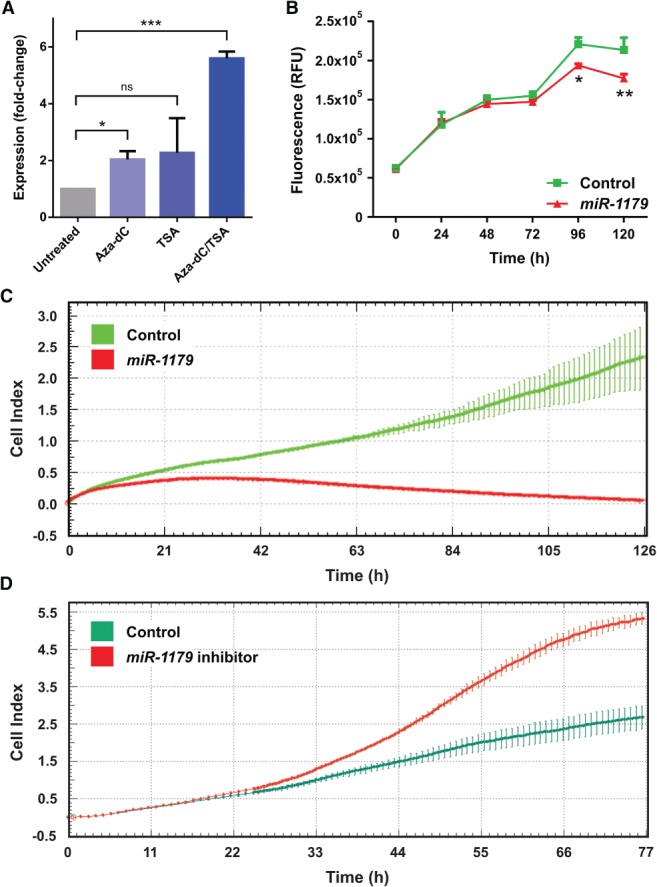
Effect of epigenetically active drugs on miR‐1179 gene expression and of miR‐1179 transfection on cell viability/proliferation of NSCLC cells. (A) A549 cells were treated with either Aza‐dC, TSA, or a combination of Aza‐dC and TSA. miR‐1179 gene expression was found to be upregulated in drug‐treated cells, as determined by RT‐PCR. The fold change in expression of drug‐treated cells as compared with untreated cells is shown. *p < 0.05; ***p < 0.001; ns, not significant. (B) Reduced viability of miR‐1179 mimic‐transfected NCI‐H2073 cells as compared with control cells was observed. Experiments were performed in triplicate. Error bars indicate standard deviations. RFU, relative fluorescence units; *p < 0.05; **p < 0.01. (C) Reduced proliferation of miR‐1179 mimic‐transfected NCI‐H2073 cells as compared with controls was found with the xCELLigence RTCA system. Cells were plated in triplicate. Error bars indicate standard deviations. (D) Increased proliferation of stably miR‐1179‐transfected A549 cells treated with miR‐1179 inhibitors as compared with controls was found with the xCELLigence RTCA system. Cells were plated in triplicate. Error bars indicate standard deviations. Cell index, quantitative measure of cell number.

To investigate whether methylation does indeed contribute to transcriptional regulation of the miR‐1179 gene, we treated A549 cells with Aza‐dC and/or TSA, and used RT‐PCR to compare miR‐1179 gene expression before and after drug treatment. Statistically significant upregulation of miR‐1179 gene expression after Aza‐dC and Aza‐dC/TSA treatment was observed (Figure 5A). In addition, reduced methylation and increased histone H4 acetylation of the miR‐1179 gene were found after drug treatment (supplementary material, Figure S10).

Moreover, we investigated whether miR‐1179 gene overexpression leads to suppressed growth of NSCLC cells. Accordingly, we used a biochemical assay to measure cell viability as followed by cell proliferation of NCI‐H2073 cells transfected with either miR‐1179 mimics or with random sequence miRNA mimic controls. We observed statistically significant reductions of the viability of cells transfected with miR‐1179 mimics as compared with control cells after both 96 h and 120 h (*p* = 0.018 and *p* = 0.002, respectively; Figure 5B). In addition, we measured cell proliferation in real time, and detected significantly reduced proliferation rates of cells transfected with miR‐1179 mimics as compared with cells transfected with a control miRNA mimic (Figure 5C). The opposite effect was seen when we transfected miR‐1179 inhibitors into cells that stably express miR‐1179 (Figure 5D). Overall, these results indicate that miR‐1179 has tumour cell growth‐suppressing properties in NSCLC cells.

## Discussion

Downregulated miRNA expression caused by methylation of genes encoding these miRNAs may be involved in the pathogenesis of NSCLCs 4, 5, 6, 8, 30, 31. We reported previously that epigenetically active drugs can upregulate the expression of certain methylated miRNA genes in NSCLC cells. Moreover, we demonstrated that genes encoding miR‐9‐3 and miR‐193a are frequently tumour‐specifically methylated in NSCLC patients 8. However, until now, only a few methylated miRNA genes in NSCLCs have been known. Thus, to obtain extensive information about methylation changes of miRNA genes in NSCLC patients, in this study we performed a high‐throughput search for methylated miRNA genes in TU samples and NL samples of 50 NSCLC patients by the use of MeDIP‐chip analyses. Overall, we identified 34 unique miRNA genes with increased methylation in these TU samples. For most of them, it was previously unknown whether they may be methylated, but for some others (e.g. those encoding members of the miR‐9 and the miR‐124 families) it has already been reported that they may be methylated in NSCLCs 32, 33, 34. For instance, Lujambio *et al* 32 reported an association between tumour‐specific miR‐9‐3 gene methylation and the appearance of lymph node metastases in NSCLC patients. Methylation of the genes encoding miR‐124‐1 and miR‐124‐2 was found to be correlated with shorter OS of NSCLC patients in the study by Kim *et al* 33. In addition, Tellez *et al* 34 investigated miR‐196b gene methylation in sputum samples from stage I–III NSCLC patients and from control individuals, and reported that detection of miR‐196b gene methylation was strongly associated with lung cancer diagnosis.

Several oncogenic miRNA genes (e.g. those encoding miR‐25, miR‐93, and miR‐106b) have been reported to be upregulated in NSCLCs 35. However, hypomethylation was identified as mechanism for upregulation of only a few miRNA genes (e.g. those encoding miR‐224 and let‐7a‐3) [36,37]. For none of the 15 miRNA genes with increased methylation in NL samples identified in our study has potential regulation by methylation been reported so far. Moreover, the vast majority of them are functionally uncharacterized, and their role in tumourigenesis needs to be investigated.

To confirm our results obtained by MeDIP‐chip analyses, we additionally determined the methylation of selected miRNA genes by use of the gene‐specific approach MS‐HRM in a larger number of TU samples and NL samples. These specimens were characterized during the years 2000–2004 by pathologists who are not involved in the current study, and according to criteria that are not completely updated with respect to the current classification of lung tumours. MS‐HRM analyses were performed for relatively well‐known miRNA genes as well as for relatively unknown miRNA genes in malignant diseases. Our data revealed that methylation of all selected miRNA genes was statistically significantly higher in TU samples than in NL samples, which confirmed our MeDIP‐chip data. In addition, these findings indicate that the tumour specimens contained a sufficient number of malignant cells, which confirms the appropriateness of the samples used. Moreover, ROC curve analyses of MS‐HRM results clearly distinguished between TU samples and NL samples. Interestingly, the miR‐10b gene was identified as the strongest discriminator between sample types, with an AUC of 0.93. In the literature, the role of the miR‐10b gene in NSCLCs and in other cancers is controversial. Whereas some authors have reported that the miR‐10b gene may act as a TSG in gastric cancer [38,39], others have suggested an oncogenic function of the miR‐10b gene in breast, hepatocellular and colorectal carcinomas, as well as in NSCLCs and gliomas 40, 41, 42, 43, 44, 45. In addition, a role of miR‐10b in metastasis by promoting the migration and invasion of breast cancer cells has been described [40,45]. However, in the pathogenesis of NSCLCs, the role of miR‐10b gene methylation needs to be further investigated.

When we compared our miRNA methylation data with clinicopathological characteristics of NSCLC patients, we found that the miR‐129‐2 gene was more frequently methylated in stage III than in stage I/II patients. miR‐129‐2 is a tumour cell growth‐suppressing miRNA in NSCLC and other malignancies, and its expression is regulated by methylation of the gene encoding it [26,46]. Recently, Torres‐Ferreira *et al* 47 demonstrated that a high level of miR‐129‐2 gene methylation is associated with shorter DFS of prostate cancer patients, and Liu *et al* 46 observed an association between low miR‐129‐2 gene expression in TUs and shorter OS and DFS of hepatocellular carcinoma patients. However, it needs to be determined whether miR‐129‐2 gene expression/methylation has a potential prognostic impact in NSCLC patients.

It was reported previously that the methylation pattern of certain genes may differ between adenocarcinomas and squamous cell carcinomas of the lung. Whereas the frequencies of *RASSF1A* and *APC* methylation were higher in adenocarcinomas, the frequency of *p16* methylation was higher in squamous cell carcinomas [48,49]. However, for many other genes, no differences in the methylation frequencies between these two NSCLC subtypes were seen 23. In our analyses, we did not find such differences, indicating that methylation of the six miRNA genes is not specific for either adenocarcinomas or squamous cell carcinomas of the lung.

miRNA target prediction with bioinformatic approaches revealed potential miRNA–mRNA interactions that may be important for tumour development. Predicted miRNA targets included several mRNAs encoding proteins that are involved in WNT signalling (e.g. *FGF9* and *CTNND1*), cell cycle regulation (e.g. *CDK6*, *E2F5*, and *E2F6*), and MAPK signalling (e.g. *MAPK10*, *MAP3K9*, and *PRKCA*). Because the role of miR‐1179 in NSCLCs was previously unknown, and our *in silico* target prediction indicated a potential function of miR‐1179 in cell cycle regulation, proliferation‐promoting signalling pathways, or apoptosis, we selected this miRNA for further analyses. One of the predicted targets is cyclin E1, a protein that forms a complex with CDK2 and is involved in positive cell cycle regulation. Cyclin E expression is upregulated in several tumour types, and an association between high *CCNE1* expression and disease progression/poor prognosis of patients with certain tumour types has been reported [27,28]. Upregulation of *CCNE1* expression in tumour samples was also seen when we analysed lung adenocarcinoma and lung squamous cell carcinoma datasets from the TCGA database. In *in vitro* experiments, we detected deregulated *CCNE1* expression in NSCLC cells transfected with miR‐1179 mimics or miR‐1179 inhibitors as compared with control cells. In addition, we observed that miR‐1179 binds to the regulatory sequence of *CCNE1*, which may explain the tumour cell growth‐inhibitory effects. The miR‐1179*–CCNE1* association needs to be elucidated in future studies.

On the basis of our data, we hypothesized that the miR‐1179 gene may function as a TSG in NSCLCs. In *in vitro* experiments using NSCLC cell line models, we detected upregulated miR‐1179 gene expression after treatment of cells with epigenetically active drugs, indicating that expression of this miRNA is indeed affected by methylation. Moreover, we observed reduced cell viability and cell proliferation of miR‐1179 mimic‐transfected NSCLC cells. Loss‐of‐function experiments with miR‐1179 inhibitors revealed increased cell proliferation, supporting the hypothesis that the miR‐1179 gene may function as a TSG in NSCLCs. However, in the literature, the role of miR‐1179 in tumourigenesis is controversial. Whereas, in papillary thyroid tumours, miR‐1179 expression was found to be frequently downregulated, it was found to be upregulated in oesophageal squamous cell carcinomas 50. Additionally, it has been reported that, in oesophageal squamous cell carcinoma cells, miR‐1179 inhibition leads to decreased invasion of these cells as compared with control cells 51. A potential explanation for these controversial findings might be that the function of miR‐1179 depends on the tumour type. Recently, the miR‐1179 gene was identified as a TSG in glioblastomas *in vivo* 52. Additional studies, in particular tumour‐xenograft experiments in mice, are necessary to further elucidate the potential tumour‐suppressive role of miR‐1179 in NSCLCs and in other malignant diseases.

Overall, using a high‐throughput approach to investigate the methylation of miRNA genes in a large number of NSCLC patients, we identified 34 miRNA genes with increased methylation in TU samples. For many of them, transcriptional regulation by methylation in NSCLCs was previously unknown. We observed that some of these miRNAs are involved in certain molecular pathways. In addition, our data suggest that miR‐1179 may be an epigenetically regulated, putative tumour cell growth suppressor in NSCLCs. In summary, the results of our study stress the importance of methylation of miRNA genes for the pathogenesis of NSCLCs.

## Author contributions statement

GH and SZM designed the study. GH, CA, TT, ET and BZ performed experiments. GH, IS, ET and SZ analysed data and generated the figures. GL, AEP, BD, BMA, WK and CCZ provided tissue samples and clinical data. GH and SZM wrote the manuscript. All authors gave final approval to the submitted and published versions.

**SUPPLEMENTARY MATERIAL ONLINE**

**Supplementary materials and methods**

**Supplementary figure legends**

**Figure S1.** Boxplots of MeDIP‐chip probes before and after normalization

**Figure S2.** MS‐HRM assays for *miR‐10b*, *miR‐129‐2*, *miR‐137*, *miR‐572*, *miR‐1179* and *miR‐3150b*

**Figure S3.** A Venn diagram demonstrating the overlap of predicted mRNA targets of *miR‐10b*, *miR‐129‐2*, *miR‐137*, *miR‐572*, *miR‐1179* and *miR‐3150b*

**Figure S4.** Representation of top predicted targets of *miR‐10b* and their relation to certain molecular pathways

**Figure S5.** Representation of top predicted targets of *miR‐129‐2* and their relation to certain molecular pathways

**Figure S6.** Representation of top predicted targets of *miR‐137* and their relation to certain molecular pathways

**Figure S7.** Representation of top predicted targets of *miR‐3150* and their relation to certain molecular pathways

**Figure S8.** Representation of the two predicted targets of *miR‐572* and their relation to certain molecular pathways

**Figure S9.** *CCNE1* expression in TU and NL samples of NSCLC patients and effect of *CCNE1* expression on overall survival (OS) of NSCLC patients

**Figure S10.** Effect of Aza‐dC and/or TSA on methylation and histone acetylation in A549 cells

**Table S1.** Clinico‐pathological characteristics of 50 NSCLC patients used for MeDIP‐chip analyses

**Table S2.** Primer sequences for MS‐HRM and ChIP analyses

**Table S3.** Tumour‐specifically methylated miRNA‐encoding genes identified by MeDIP‐chip analyses

**Table S4.** MiRNA‐encoding genes (n = 15) with increased methylation in NL compared to TU identified by MeDIP‐chip analyses

**Table S5.** Methylation values of 6 miRNA‐encoding genes in TU and NL samples of 104 NSCLC patients determined by MS‐HRM analyses

**Table S6.** Comparison of MS‐HRM data from 6 miRNA‐encoding genes with certain clinico‐pathological characteristics from 104 NSCLC patients

**Table S7.** Predicted targets of *miR‐10b, miR‐129‐2, miR‐137, miR‐572, miR‐1179* and *miR‐3150* identified by miRDB, miRanda, miRMap, RNAhybrid and Targetscan

## Supporting information


**Appendix S1** Supplementary Materials and Methods

**Figure S1.** Boxplots of MeDIP‐chip probes before and after normalization. For the raw data, the log2 ratios were taken and the median of all replicates per probe was calculated. All non‐malignant lung tissue samples and primary tumour samples were patient‐matched.

**Figure S2.** MS‐HRM assays for miR‐10b, miR‐129‐2, miR‐137, miR‐572, miR‐1179 and miR‐3150b. HMR plots, regression lines and linear equations are shown. Colour code: black, 100% methylated; pink, 75% methylated; orange, 50% methylated; blue, 25% methylated; red, 10% methylated; green, 0% methylated.

**Figure S3.** A Venn diagram demonstrating the overlap of predicted mRNA targets of miR‐10b, miR‐129‐2, miR‐137, miR‐572, miR‐1179 and miR‐3150b.

**Figure S4.** Representation of top predicted targets of miR‐10b and their relation to certain molecular pathways. Targets are ranked based on their prediction score from red (highest score) to light blue (lowest score).

**Figure S5.** Representation of top predicted targets of miR‐129‐2 and their relation to certain molecular pathways. Targets are ranked based on their prediction score from red (highest score) to light blue (lowest score).

**Figure S6.** Representation of top predicted targets of miR‐137 and their relation to certain molecular pathways. Targets are ranked based on their prediction score from red (highest score) to light blue (lowest score).

**Figure S7.** Representation of top predicted targets of miR‐3150 and their relation to certain molecular pathways. Targets are ranked based on their prediction score from red (highest score) to light blue (lowest score).

**Figure S8.** Representation of the two predicted targets of miR‐572 and their relation to certain molecular pathways. Targets are ranked based on their prediction score from red (highest score) to light blue (lowest score).

**Figure S9.** CCNE1 expression in TU and NL samples of NSCLC patients and effect of CCNE1 expression on overall survival (OS) of NSCLC patients. **(A)** Publicly available RNA‐seq data of the TCGA datasets LUAD (lung adenocarcinomas) and **(B)** LUSC (lung squamous cell carcinomas) were analysed for expression of CCNE1 in NL and in TU samples of > 1.000 NSCLC patients. Each dot represents a single tissue sample. ***, p‐value < 0.0001; NL, non‐malignant lung tissue; TU, primary non‐small cell lung cancer tissue. **(C)** CCNE1 expression determined by RNA‐sequencing was compared with OS of 492 lung adenocarcinoma patients and **(D)** 488 lung squamous cell carcinoma patients from the TCGA database using the online tool OncoLnc (http://www.oncolnc.org/). **(E)** CCNE1 expression determined by Affymetrix microarray analyses was compared with OS of 720 lung adenocarcinoma patients and **(F)** 524 lung squamous cell carcinoma patients using the online tool KM plotter (http://kmplot.com). LUAD, lung adenocarcinoma dataset; LUSC, lung squamous cell carcinoma dataset; HR, hazard ratio.

**Figure S10.** Effect of Aza‐dC and/or TSA on methylation and histone acetylation in A549 cells. **(A)** Reduced miR‐1179 methylation in Aza‐dC treated (red) compared to untreated A549 cells determined by MS‐HRM analysis is shown. **(B)** A strong increase of histone H4 acetylation in Aza‐dC/TSA treated A549 cells is illustrated. AB, antibody; Aza‐dC, 5‐aza‐2'‐deoxycytidine; TSA, trichostatin A.

**Table S1.** Clinico‐pathological characteristics of 50 NSCLC patients used for MeDIP‐chip analyses

**Table S2.** Primer sequences for MS‐HRM and ChIP analyses

**Table S3.** Tumour‐specifically methylated miRNA‐encoding genes identified by MeDIP‐chip analyses

**Table S4.** MiRNA‐encoding genes (n = 15) with increased methylation in NL compared to TU identified by MeDIP‐chip analyses

**Table S5.** Methylation values of 6 miRNA‐encoding genes in TU and NL samples of 104 NSCLC patients determined by MS‐HRM analyses.

**Table S6.** Comparison of MS‐HRM data from 6 miRNA‐encoding genes with certain clinico‐pathological characteristics from 104 NSCLC patients. P‐values are shown.

**Table S7.** Predicted targets of *miR‐10b, miR‐129‐2, miR‐137, miR‐572, miR‐1179* and *miR‐3150* identified by miRDB, miRanda, miRMap, RNAhybrid and Targetscan. Target scores from miRDB are shown.
