# Supplementary material for: DNA methylation of microRNA‐coding genes in non‐small‐cell lung cancer patients
Source: J Pathol. 2018 Jun 20;245(4):387–98. doi: 10.1002/path.5079 (PMC6055722; doi:10.1002/path.5079)
Supplement: Supplementary file 1 — Appendix S1 Supplementary Materials and Methods [file PATH-245-387-s004.docx]

**Supplementary Materials and Methods**

Reference numbers refer to the main text list.

MeDIP-chip analyses

MeDIP was performed as described previously [23]. In brief, RNAse treated genomic DNA was sonicated using Bioruptor^TM^ (Diagenode, Liege, Belgium), denatured and immunoprecipitated using a monoclonal anti-5-methylcytosine antibody (Eurogentec, Seraing, Belgium) and protein A agarose beads (Millipore, Bedford, MA, USA) blocked with salmon sperm DNA. MeDIP samples were sent to NimbleGen service laboratories for quality control, sample labelling and hybridization to a custom designed tiling microarray. Genomic sequences for custom microarray design were obtained from USCS genome browser (assembly “GRCh37/hg19”, track “sno/miRNA”, 2000 bp upstream and 1000 bp downstream of the transcription start site) and from miRBase (version 18) database. Each microarray contained 95.082 (x6 replicates) probes, 1.375 negative controls, 340 non CG - Controls, and 2.185 positive controls (574.392 probes in total). Overall, probes on the microarrays covered 1552 miRNA genes and 390 snoRNA genes. Arrays were scanned on a GenePix 4200A Scanner using GenePix 6.0 Software and data were extracted from scanned images by using NimbleScan 2.3 extraction software (NimbleGen Systems, Inc., Mannheim, Germany). Microarray design and MeDIP-chip data were deposited in the GEO database (accession no. GSE86173).

Statistical analyses

NimbleGen microarray data from clinical samples were normalized with NimbleGen-normalization (Tukey’s biweight mean across each sample’s log2 ratios are computed and subtracted from the individual log2 ratios). For all analyses the median of all replicates per probe was used. Quality assessment was done by plotting boxplots for each tissue sample. To test for potential differences in methylation between TU and NL samples, we calculated paired t-statistics with permutation adjusted p-values for step down multiple testing (R-function mt.maxT) using the normalized data which provided strong control of the family-wise error rate (FWER). For all analyses the significance level was set to 0.1. These analyses were conducted with the statistics software R 2.14.1 and the R-package multtest [53,54]. Heatmaps and principal component (PCA) plots were generated using clustVis [55]. CpG island search was performed using the online tool *cpg_islands* from www.bioinformatics.org (default settings: Obs/Exp value > 0.6 and GC content > 50%).

To calculate methylation differences between TU and NL samples obtained by MS-HRM analyses, Wilcoxon signed rank tests were used. Receiver operating characteristic (ROC) curve analysis was performed using GraphPad Prism 6 software. Two sided p-values derived from Wilcoxon signed rank tests and ROC curve analyses were Bonferroni corrected and an adjusted p-value < 0.05 was considered as statistically significant.

miRNA target prediction was performed using miRDB and miRanda, miRMap, RNAhybrid and Targetscan algorithms from miRWalk2.0 [56,57]. Only miRNA target genes which were predicted by all 5 algorithms were selected for subsequent analyses. The R package GOplot was used for data visualization [58].

MS-HRM data of six miRNA-encoding genes were compared with clinico-pathological characteristics (gender, age, histology, tumour stage, lymph node stage, stage of disease, disease recurrence, DFS and OS) of NSCLC patients in an exploratory analysis. For survival analyses TU/NL methylation ratios were calculated and patients with a TU/NL ratio >1.5 were considered as methylated. Chi^2^ tests / Fisher´s exact tests were used to calculate differences between groups and t-tests / Mann-Whitney U tests were used to calculate differences between means. Survival analyses of patients were performed using log rank testing. A p-value < 0.05 was considered as statistically significant. Similar tests were applied to compare *CCNE1* expression with clinico-pathological characteristics of LUAD and LUSC datasets. All analyses were performed using the statistics software GraphPad Prism (version 6). The online tools OncoLnc [59] and KM plotter [60] were used for survival analyses in publically available datasets.
