# Supplementary figures and images for: DNA methylation of microRNA‐coding genes in non‐small‐cell lung cancer patients
Source: J Pathol. 2018 Jun 20;245(4):387–98. doi: 10.1002/path.5079 (PMC6055722; doi:10.1002/path.5079)

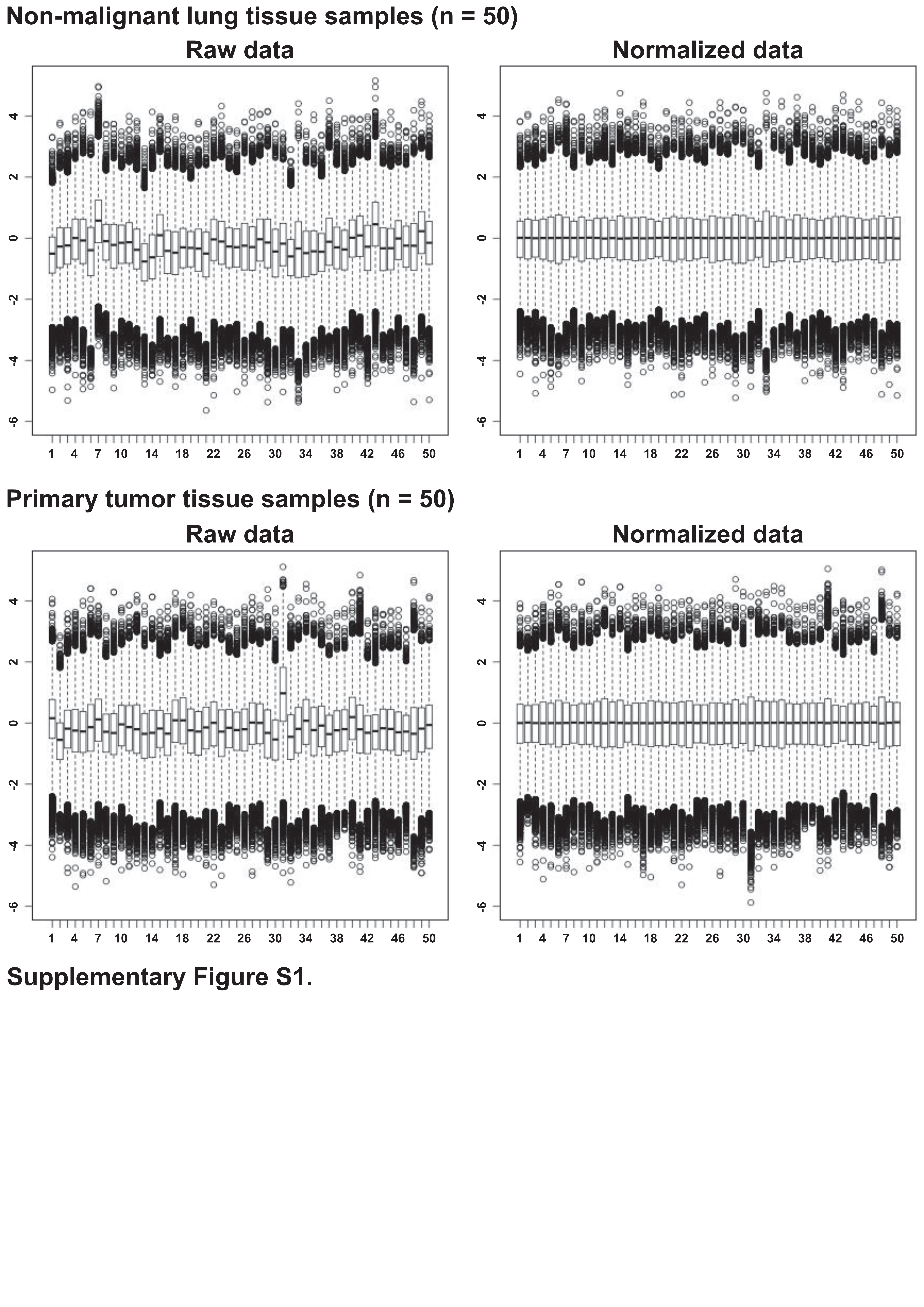

Supplement: Supplementary file 2 — Figure S1. Boxplots of MeDIP‐chip probes before and after normalization. For the raw data, the log2 ratios were taken and the median of all replicates per probe was calculated. All non‐malignant lung tissue samples and primary tumour samples were patient‐matched. [file PATH-245-387-s009.tiff]

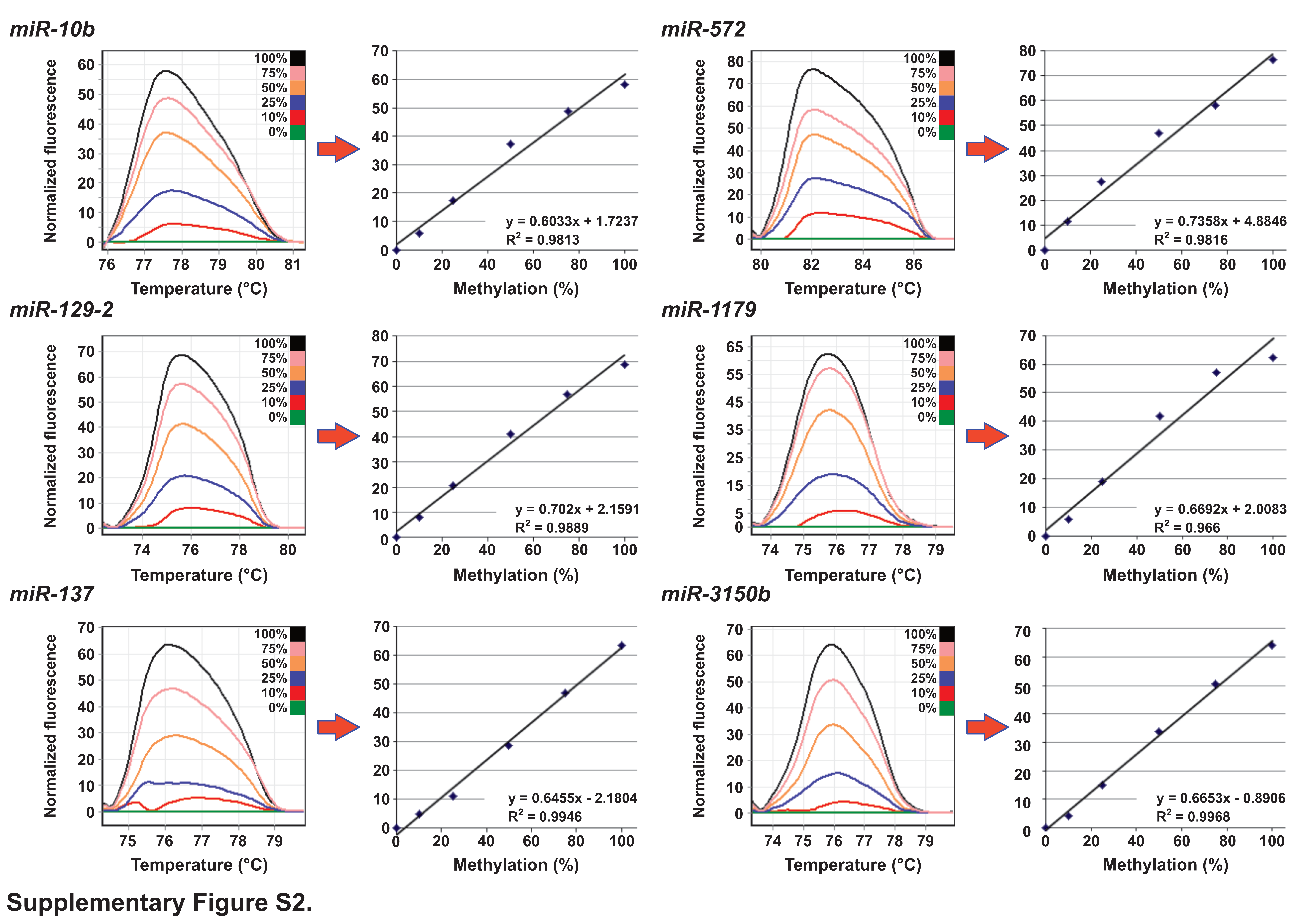

Supplement: Supplementary file 3 — Figure S2. MS‐HRM assays for miR‐10b, miR‐129‐2, miR‐137, miR‐572, miR‐1179 and miR‐3150b. HMR plots, regression lines and linear equations are shown. Colour code: black, 100% methylated; pink, 75% methylated; orange, 50% methylated; blue, 25% methylated; red, 10% methylated; green, 0% methylated. [file PATH-245-387-s011.tiff]

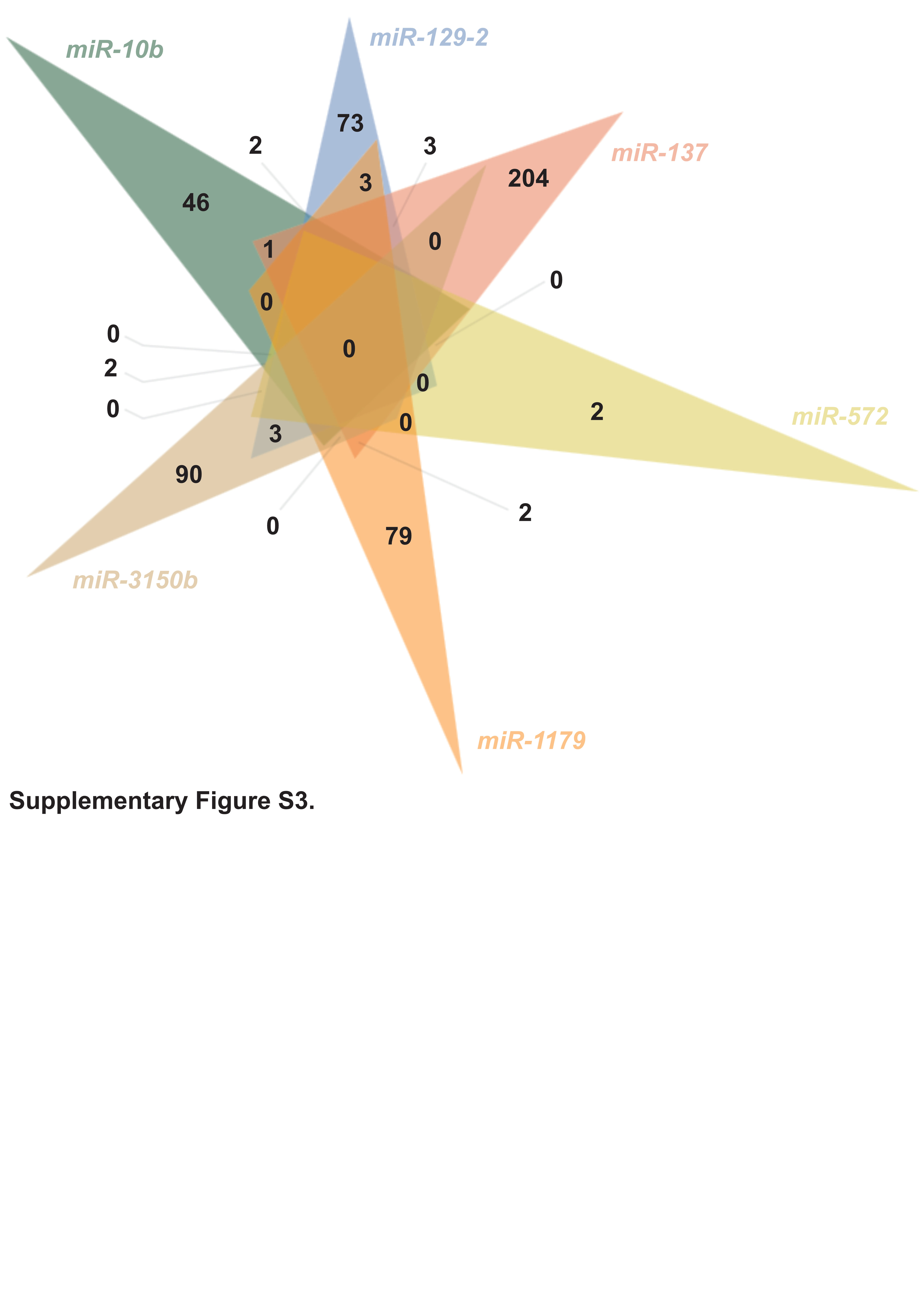

Supplement: Supplementary file 4 — Figure S3. A Venn diagram demonstrating the overlap of predicted mRNA targets of miR‐10b, miR‐129‐2, miR‐137, miR‐572, miR‐1179 and miR‐3150b. [file PATH-245-387-s016.tiff]

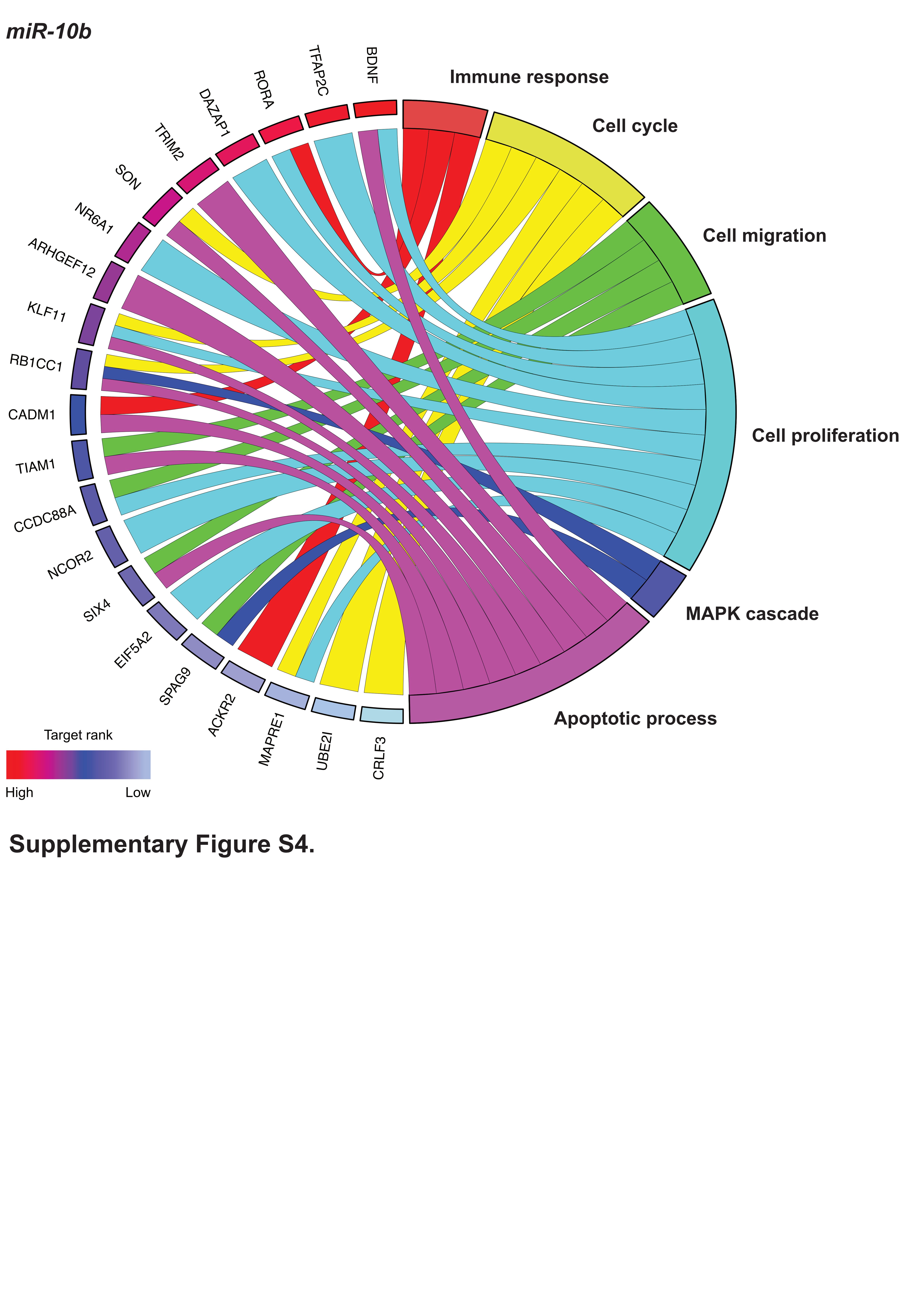

Supplement: Supplementary file 5 — Figure S4. Representation of top predicted targets of miR‐10b and their relation to certain molecular pathways. Targets are ranked based on their prediction score from red (highest score) to light blue (lowest score). [file PATH-245-387-s008.tiff]

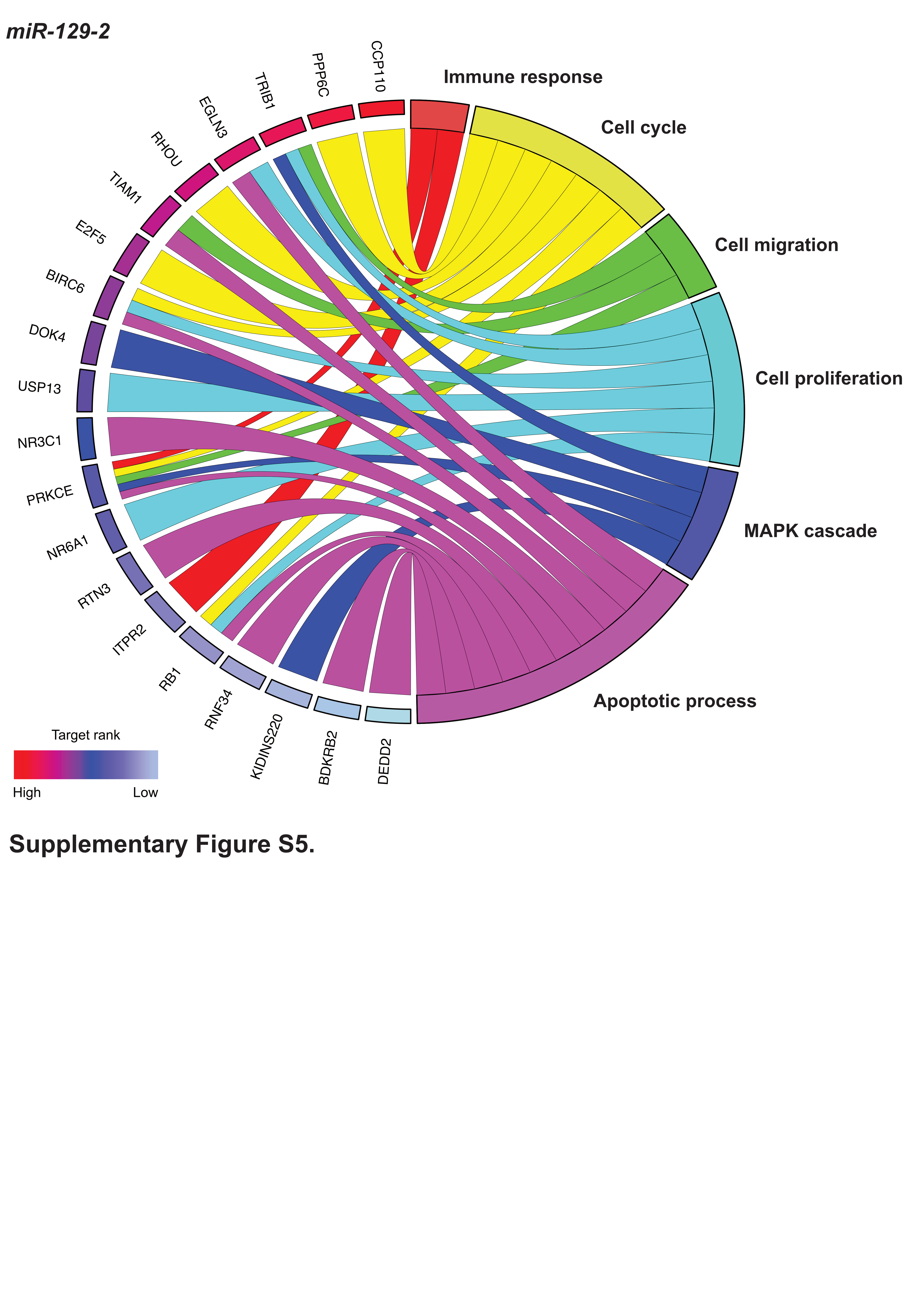

Supplement: Supplementary file 6 — Figure S5. Representation of top predicted targets of miR‐129‐2 and their relation to certain molecular pathways. Targets are ranked based on their prediction score from red (highest score) to light blue (lowest score). [file PATH-245-387-s005.tiff]

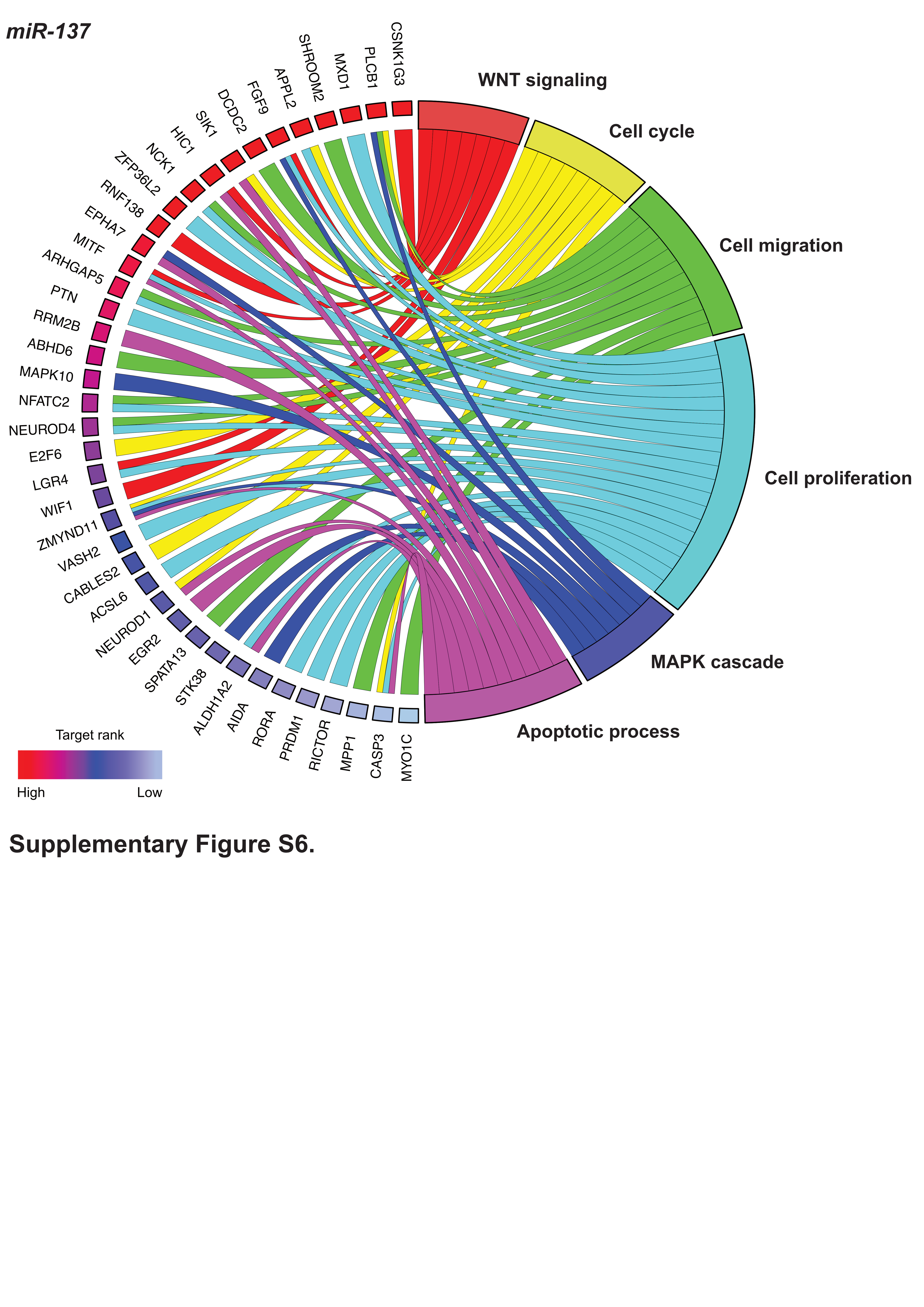

Supplement: Supplementary file 7 — Figure S6. Representation of top predicted targets of miR‐137 and their relation to certain molecular pathways. Targets are ranked based on their prediction score from red (highest score) to light blue (lowest score). [file PATH-245-387-s018.tiff]

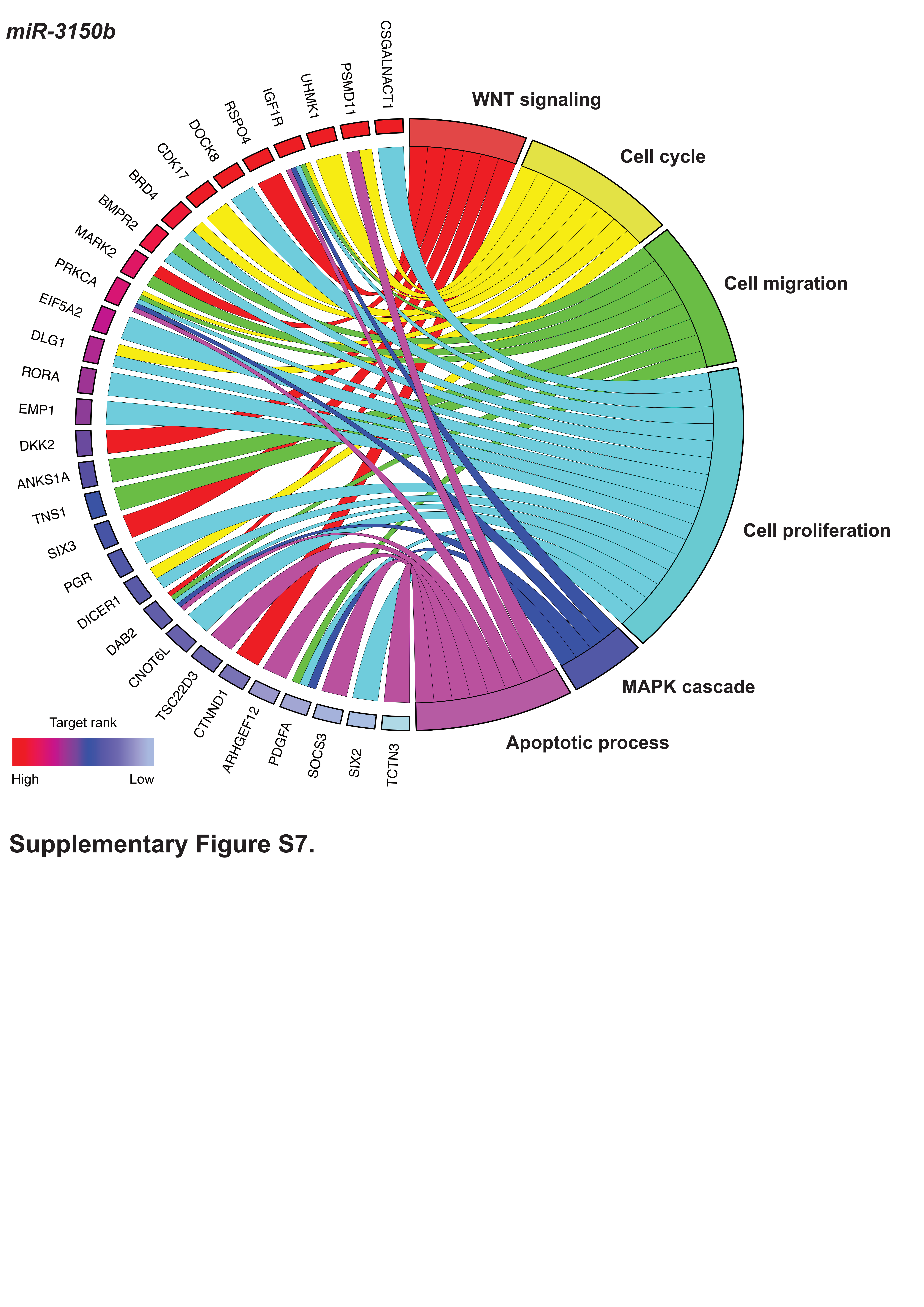

Supplement: Supplementary file 8 — Figure S7. Representation of top predicted targets of miR‐3150 and their relation to certain molecular pathways. Targets are ranked based on their prediction score from red (highest score) to light blue (lowest score). [file PATH-245-387-s003.tiff]

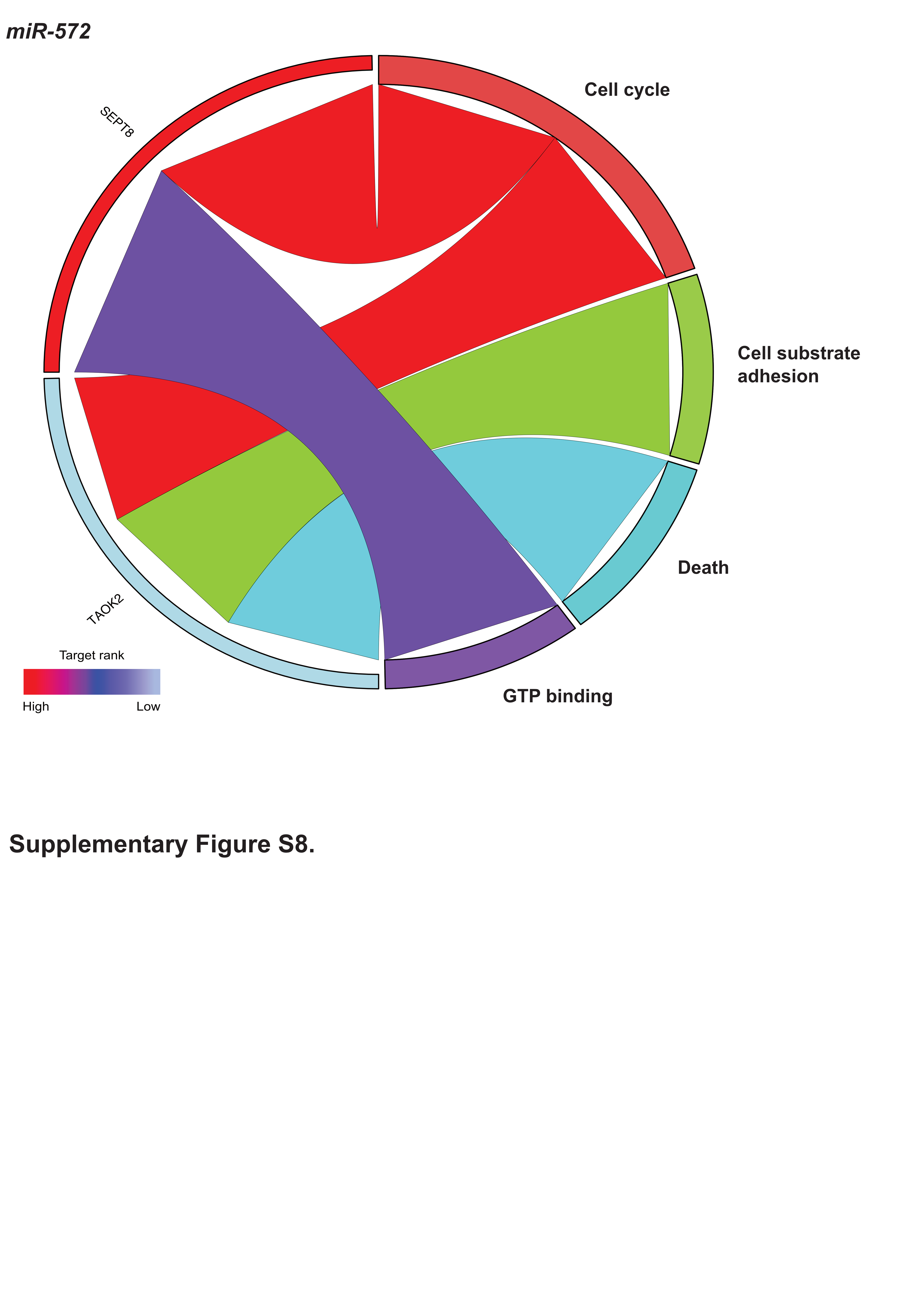

Supplement: Supplementary file 9 — Figure S8. Representation of the two predicted targets of miR‐572 and their relation to certain molecular pathways. Targets are ranked based on their prediction score from red (highest score) to light blue (lowest score). [file PATH-245-387-s015.tiff]

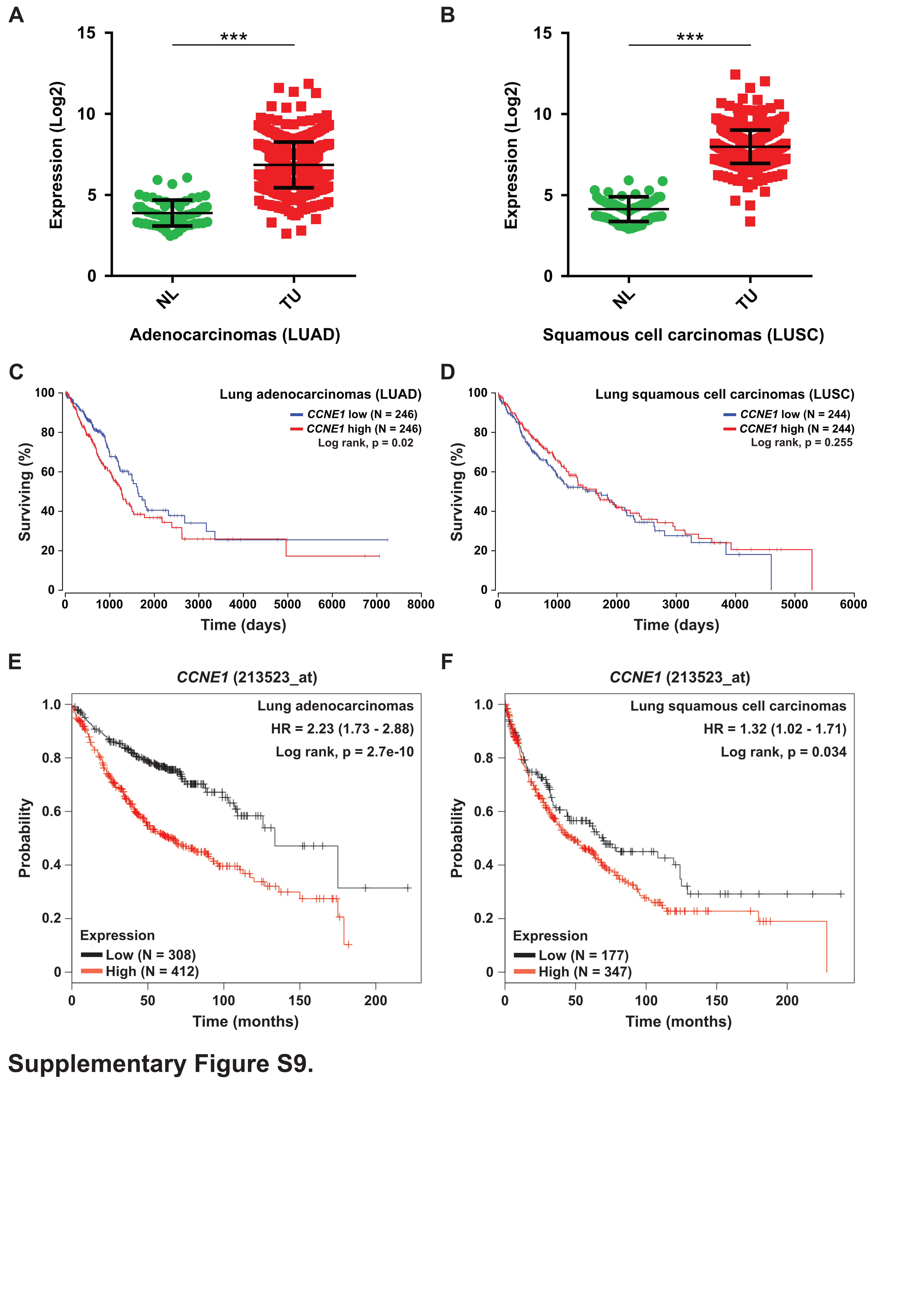

Supplement: Supplementary file 10 — Figure S9. CCNE1 expression in TU and NL samples of NSCLC patients and effect of CCNE1 expression on overall survival (OS) of NSCLC patients. (A) Publicly available RNA‐seq data of the TCGA datasets LUAD (lung adenocarcinomas) and (B) LUSC (lung squamous cell carcinomas) were analysed for expression of CCNE1 in NL and in TU samples of > 1.000 NSCLC patients. Each dot represents a single tissue sample. ***, p‐value < 0.0001; NL, non‐malignant lung tissue; TU, primary non‐small cell lung cancer tissue. (C) CCNE1 expression determined by RNA‐sequencing was compared with OS of 492 lung adenocarcinoma patients and (D) 488 lung squamous cell carcinoma patients from the TCGA database using the online tool OncoLnc (http://www.oncolnc.org/). (E) CCNE1 expression determined by Affymetrix microarray analyses was compared with OS of 720 lung adenocarcinoma patients and (F) 524 lung squamous cell carcinoma patients using the online tool KM plotter (http://kmplot.com). LUAD, lung adenocarcinoma dataset; LUSC, lung squamous cell carcinoma dataset; HR, hazard ratio. [file PATH-245-387-s012.tiff]

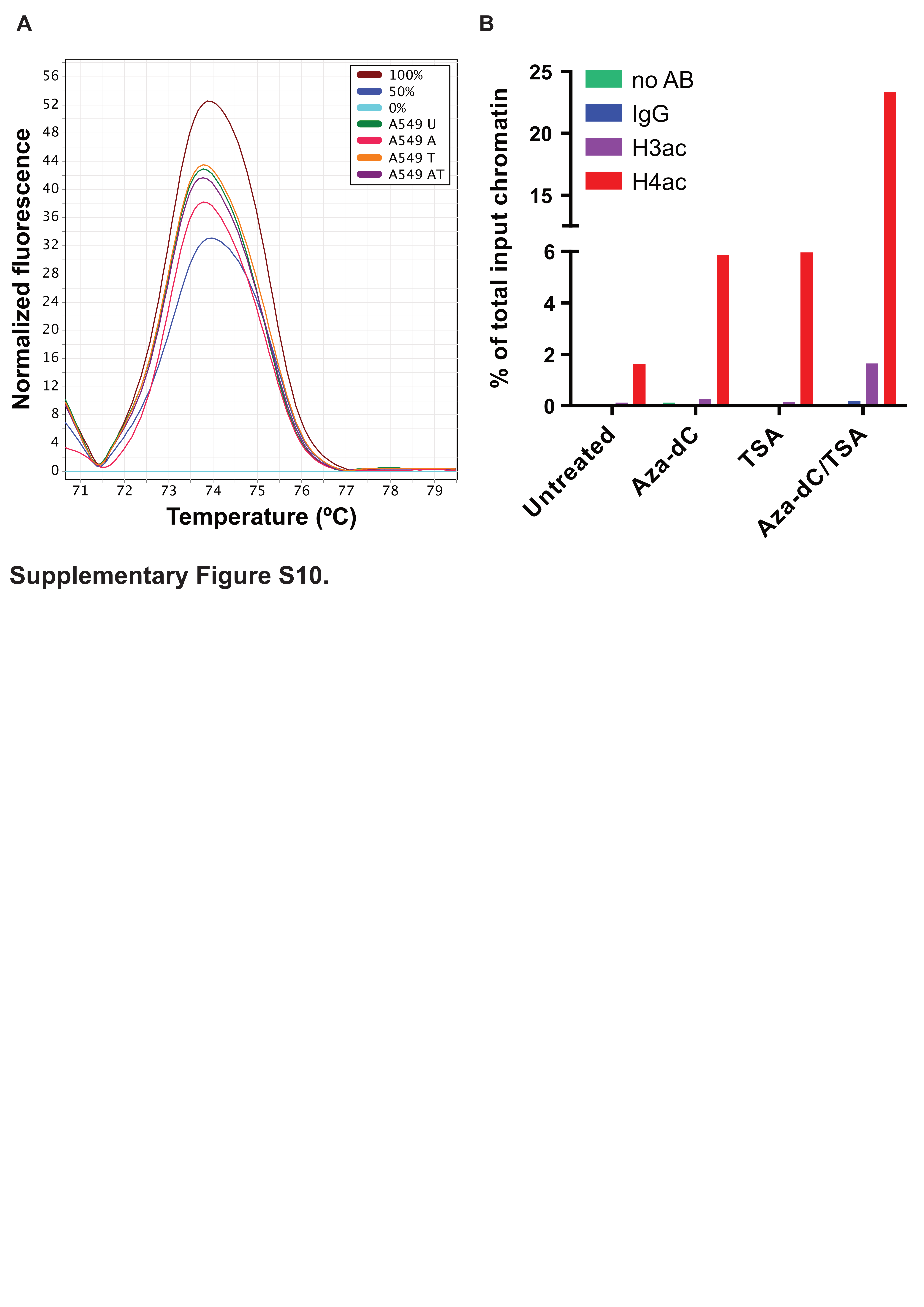

Supplement: Supplementary file 11 — Figure S10. Effect of Aza‐dC and/or TSA on methylation and histone acetylation in A549 cells. (A) Reduced miR‐1179 methylation in Aza‐dC treated (red) compared to untreated A549 cells determined by MS‐HRM analysis is shown. (B) A strong increase of histone H4 acetylation in Aza‐dC/TSA treated A549 cells is illustrated. AB, antibody; Aza‐dC, 5‐aza‐2'‐deoxycytidine; TSA, trichostatin A. [file PATH-245-387-s002.tiff]
