# Supplementary material for: DNA methylation of microRNA‐coding genes in non‐small‐cell lung cancer patients
Source: J Pathol. 2018 Jun 20;245(4):387–98. doi: 10.1002/path.5079 (PMC6055722; doi:10.1002/path.5079)
Supplement: Supplementary file 12 — Table S1. Clinico‐pathological characteristics of 50 NSCLC patients used for MeDIP‐chip analyses [file PATH-245-387-s007.docx]

**Table S1.** Clinico-pathological characteristics of 50

NSCLC patients used for MeDIP-chip analyses

Variables N

Age

≤60 22

>60 28

Gender

Male 26

Female 24

Histology

Adenocarcinoma 33

Squamous cell carcinoma 17

Disease stage

IA 22

IB 27

IIA 1

T stage

T1 23

T2 27

N stage

N0 49

N1 1

Disease recurrence

No 29

Yes 21

NSCLC, non-small cell lung cancer
